# Supplementary material for: Nanoencapsulation of amitriptyline enhances the potency of antidepressant-like effects and exhibits anxiolytic-like effects in Wistar rats
Source: PLoS One. 2025 Feb 28;20(2):e0316389. doi: 10.1371/journal.pone.0316389 (PMC11870345; doi:10.1371/journal.pone.0316389)
Supplement: Attachment — Submitted filename: rebuttal letter- Valadez-Lemus.docx [file pone.0316389.s006.docx]

November 6, 2024

Dear Editor and Reviewers,

We would like to express our gratitude for the time and effort dedicated to improving this manuscript. We have addressed all of your questions and suggestions, which we believe have substantially improved the manuscript. Briefly, the main changes are as follows:

- We conducted new experiments using the Marble Buring test to asses anxiety-like behaviour in rodents.
- We create a new figure (Fig. 9) to illustrate these new results.
- All sections of the manuscript have been revised to incorporate these findings.
- The English language has been carefully edited to correct any grammatical or spelling errors.
- The introduction section was revised to highlight nanoparticles as a promising pharmacological strategy.
- The Method section was expanded to provide further details on the methodological approaches, including the evaluation of the nanoparticles, identification of estrous phases in the rat cytology, and rationale behind the dose-response curve for each compound analyzed.
- Tables were included into the main text as required.
- The Discussion section was revised to reflect Reviewer # 2 suggestions.
- The title of the study has been changed, as we consider the new results provide evidence supporting this modification.
- Additional supporting material containing the raw data has been uploaded to reinforce our findings.

Bellow, you will find our responses to your specific questions (highlighted in green). Please note that the page and line numbers correspond to the clean version of the manuscript, titled “Manuscript Valadez-Lemus”.

1. When submitting your revision, we need you to address these additional requirements.

Please ensure that your manuscript meets PLOS ONE's style requirements, including those for file naming. The PLOS ONE style templates can be found at

<https://journals.plos.org/plosone/s/file?id=wjVg/PLOSOne_formatting_sample_main_body.pdf> and

<https://journals.plos.org/plosone/s/file?id=ba62/PLOSOne_formatting_sample_title_authors_affiliations.pdf>.

2. We note that the grant information you provided in the ‘Funding Information’ and ‘Financial Disclosure’ sections do not match.

When you resubmit, please ensure that you provide the correct grant numbers for the awards you received for your study in the ‘Funding Information’ section.

3. Thank you for stating the following financial disclosure:

 [CONACYT-SEP funding under Grant CB A1-S-39237.].

Please state what role the funders took in the study.  If the funders had no role, please state: ""The funders had no role in study design, data collection and analysis, decision to publish, or preparation of the manuscript.""

If this statement is not correct you must amend it as needed.

Please include this amended Role of Funder statement in your cover letter; we will change the online submission form on your behalf.

4. Please expand the acronym “CONACYT-SEP” (as indicated in your financial disclosure) so that it states the name of your funders in full.

This information should be included in your cover letter; we will change the online submission form on your behalf.

5. Thank you for stating the following in the Acknowledgments Section of your manuscript:

[The authors appreciate the technical assistance of Manuel Aguilar Franco (CFTA-UNAM) for the SEM. Ramon Valadez-Lemus acknowledge to Consejo Nacional de Ciencia y Tecnología (CONACYT) by the grant number 928892.]

R: Thank you for this information. In this part the student wanted to thank CONACYT for a doctoral fellowship. We eliminated the fellowship’s ID to avoid any misunderstanding.

We note that you have provided funding information that is not currently declared in your Funding Statement. However, funding information should not appear in the Acknowledgments section or other areas of your manuscript. We will only publish funding information present in the Funding Statement section of the online submission form.

Please remove any funding-related text from the manuscript and let us know how you would like to update your Funding Statement. Currently, your Funding Statement reads as follows:

  [CONACYT-SEP funding under Grant CB A1-S-39237.].

Please include your amended statements within your cover letter; we will change the online submission form on your behalf.

R: This information as been updated in the cover letter to the Editor

 6. Your ethics statement should only appear in the Methods section of your manuscript. If your ethics statement is written in any section besides the Methods, please move it to the Methods section and delete it from any other section. Please ensure that your ethics statement is included in your manuscript, as the ethics statement entered into the online submission form will not be published alongside your manuscript.

R: We eliminate the ethics statement from the manuscript, and we maintained it only the Methods section. Thank you.

7. We note that Figure 2 in your submission contain copyrighted images. All PLOS content is published under the Creative Commons Attribution License (CC BY 4.0), which means that the manuscript, images, and Supporting Information files will be freely available online, and any third party is permitted to access, download, copy, distribute, and use these materials in any way, even commercially, with proper attribution. For more information, see our copyright guidelines: <http://journals.plos.org/plosone/s/licenses-and-copyright>.

We require you to either (1) present written permission from the copyright holder to publish these figures specifically under the CC BY 4.0 license, or (2) remove the figures from your submission:

 R: We apologize for the misunderstanding. The Figure 2 is an original photograph captured from imaging the nanoencapsulation of amitriptyline using an electronic microscope. The image was later edited using Adobe Illustrator (arrows and scale bar). This image has not been published or presented in any proceeding of local or international meetings. If necessary, we can provide the original, unedited image.

a. You may seek permission from the original copyright holder of Figure 2 to publish the content specifically under the CC BY 4.0 license.

We recommend that you contact the original copyright holder with the Content Permission Form (<http://journals.plos.org/plosone/s/file?id=7c09/content-permission-form.pdf>) and the following text:

“I request permission for the open-access journal PLOS ONE to publish XXX under the Creative Commons Attribution License (CCAL) CC BY 4.0 (<http://creativecommons.org/licenses/by/4.0/>). Please be aware that this license allows unrestricted use and distribution, even commercially, by third parties. Please reply and provide explicit written permission to publish XXX under a CC BY license and complete the attached form.”

Please upload the completed Content Permission Form or other proof of granted permissions as an ""Other"" file with your submission.

In the figure caption of the copyrighted figure, please include the following text: “Reprinted from [ref] under a CC BY license, with permission from [name of publisher], original copyright [original copyright year].”

b. If you are unable to obtain permission from the original copyright holder to publish these figures under the CC BY 4.0 license or if the copyright holder’s requirements are incompatible with the CC BY 4.0 license, please either i) remove the figure or ii) supply a replacement figure that complies with the CC BY 4.0 license. Please check copyright information on all replacement figures and update the figure caption with source information. If applicable, please specify in the figure caption text when a figure is similar but not identical to the original image and is therefore for illustrative purposes only.

8. Please include your tables as part of your main manuscript and remove the individual files. Please note that supplementary tables (should remain/ be uploaded) as separate ""supporting information"" files".

R: Done. Thank you.

[Note: HTML markup is below. Please do not edit.]

Reviewers' comments:

Reviewer's Responses to Questions

**Comments to the Author**

1. Is the manuscript technically sound, and do the data support the conclusions?

The manuscript must describe a technically sound piece of scientific research with data that supports the conclusions. Experiments must have been conducted rigorously, with appropriate controls, replication, and sample sizes. The conclusions must be drawn appropriately based on the data presented.

Reviewer #1: Yes

Reviewer #2: Yes

2. Has the statistical analysis been performed appropriately and rigorously?

Reviewer #1: Yes

Reviewer #2: Yes

3. Have the authors made all data underlying the findings in their manuscript fully available?

The [PLOS Data policy](http://c05y1x9s.r.us-east-2.awstrack.me/L0/http:%2F%2Fwww.plosone.org%2Fstatic%2Fpolicies.action%23sharing/1/010f01919044e900-41fe1632-c19e-4c7c-8f82-38dc44f61c55-000000/Wzwjut80i6j8O34zUe5mEW-Yi6c=173) requires authors to make all data underlying the findings described in their manuscript fully available without restriction, with rare exception (please refer to the Data Availability Statement in the manuscript PDF file). The data should be provided as part of the manuscript or its supporting information, or deposited to a public repository. For example, in addition to summary statistics, the data points behind means, medians and variance measures should be available. If there are restrictions on publicly sharing data—e.g. participant privacy or use of data from a third party—those must be specified.

Reviewer #1: Yes

Reviewer #2: Yes

4. Is the manuscript presented in an intelligible fashion and written in standard English?

PLOS ONE does not copyedit accepted manuscripts, so the language in submitted articles must be clear, correct, and unambiguous. Any typographical or grammatical errors should be corrected at revision, so please note any specific errors here.

Reviewer #1: Yes

Reviewer #2: No

R: Thank you very much for your comment. We have conducted a thorough review of the manuscript’s writing, making several modifications have been made, as shown the tracked-changes document. We hope these revisions have improved the quality of the text.

5. Review Comments to the Author

Please use the space provided to explain your answers to the questions above. You may also include additional comments for the author, including concerns about dual publication, research ethics, or publication ethics. (Please upload your review as an attachment if it exceeds 20,000 characters)

Reviewer #1: Publishable article subject to minor technical corrections in language and referencing.
Authors are requested to follow the referencing style of the journal.

R: References have been revised and formatted to the journal’s requirements.

Methods and statistical plan are as per expectations.

R: Thak you very much
The need for the research and the implications, take-aways must figure in the concluding part of the introductory section.

R: Thank you very much for your helpful suggestions. We have added lines outlining the state of the art on amitriptyline nanoparticles and the need for this study (Page: 4, lines: 86-91). Additionally, a new paragraph has been included to state the study’s aims and key takeaways (Page: 5, lines: 110-118).

Reviewer #2: The manuscript presents a well-designed study investigating the potential benefits of nanoformulating amitriptyline (Ami). The authors effectively demonstrate that Ami nanoparticles (Ami-NPs) exhibit enhanced potency and efficacy in reducing immobility in the forced swimming test (FST) compared to Ami in solution.

R: Thank you very much for your kind comments.

However, the study could be strengthened by addressing the following critical questions:
Overall Critical Questions
Anxiogenic-like Effects: While the authors report that Ami-NPs mitigate certain anxiogenic-like effects in the elevated plus-maze, they do not provide a comprehensive analysis of anxiety-related behaviors. It would be valuable to include data from additional anxiety-like tests (e.g., light-dark box, Vogel test) to establish a more robust understanding of the anxiolytic properties of Ami-NPs.

R: We appreciate this suggestion. We conducted additional experiments using the marble burying test (MBT), a method used to asses general anxiety and obsessive**-**compulsive behavior (Kedia & Chattarji, 2014). The results showed a reduction in the number of buried marbles in animals treated with Ami-NPs compared to the control nanoparticles group, suggesting anxiolytic-like properties of Ami-NPs. These results have been added to the Results section (Page 18 and 19: lines: 395-409), and Figure 9. Additionally, the Methods, Discussion and Abstract sections have been updated to reflect these new results.

Mechanism of Action: The study provides evidence for enhanced bioavailability of Ami-NPs, but it lacks a deeper exploration of the underlying mechanisms. Future studies could investigate whether the nanoparticles enhance Ami's absorption, distribution, or metabolism, and how these factors contribute to the observed effects.
Long-Term Effects: The authors focus on acute administration of Ami-NPs and Ami-S. It would be interesting to evaluate the long-term effects of these formulations, including potential tolerance or dependence issues.

R: We agree with the reviewer’s insightful perspective. We have added a new subsection in the Discussion titled “Limitation of the study”, addressing the lack of clarity regarding the mechanisms of action of the Ami-loaded nanoparticles. This section discusses the increased potency of the antidepressant-like properties and emphasizes the need of further research to better understand the mechanisms of action of Ami-NPs and their long-term effects, as the results analyzed in this study were acute (page: 24, Paragraph: 541-549).

Clinical Relevance: While the preclinical findings are promising, it is essential to address the clinical relevance of these results. How might the development of Ami-NPs translate into improved patient outcomes? Could this approach potentially reduce the incidence of side effects associated with Ami therapy?

R: We appreciate this comment. We have added a new paragraph in the Discussion section, titled “Study clinical relevance”. In that section, we discuss the potential for translating our results to clinical settings and the possibility of a reducing side effects through the use of lower doses of amitriptyline (Page: 24, lines: 528-539).

Characterization of Ami-NPs: While the authors provide basic characterization data (size, polydispersity index, zeta potential, encapsulation efficiency), additional information on the stability of the nanoparticles over time and under different storage conditions would be helpful.

R: We have added lines in the Results section detailing the stability of the nanoparticles formulation (Page: 15, lines: 325-327).

Introduction Analysis
Literature review: While the introduction provides some relevant references, a more comprehensive literature review could be included to strengthen the argument for nanoencapsulation as a promising approach.
Specificity: The discussion of the benefits of nanoencapsulation could be more specific, addressing how it might improve amitriptyline's bioavailability, reduce side effects, and enhance its efficacy.
Comparison with other approaches: The introduction could briefly discuss other potential approaches to improving amitriptyline's efficacy, such as combination therapy or alternative antidepressants, to highlight the unique advantages of nanoencapsulation.

R: A new paragraph has been added to the introduction section to strengthen the argument for nanoencapsulation as a promising approach: (Page: 13, lines: 67-84)
Methodology Analysis
Justification for specific methods: The rationale behind the choice of specific nanoparticles components (PMVE/MA, Poloxamer 407) and the Box-Behnken design (BBD) for optimization could be further elaborated on.

R: The section of “*Preparation and characterization of Amitriptyline nanoparticles*”, in the Discussion section has been expanded to explain the rationale behind the election of nanoparticle components (Page: 19, lines: 418-426). Additionally, a paragraph in the Introduction section outlines the rational behind the selection of polymers used in the preparation of the Ami nanoparticles (Page: 5, lines: 96-104).

Detailed explanation of analytical methods: While the methodology mentions characterization techniques (DLS, SEM, HPLC), a more detailed explanation of how each method is used to assess particle size, morphology, and encapsulation efficiency would be beneficial.

R: Several sections of the Methods sections were revised to provide a more detailed explanation of each method used to evaluate the nanoparticle characteristics, including (Section: 2.2 “Preparation of Amitriptyline polymeric nanoparticles”; 2.3.“Optimization of the particle size of the Ami-NPs by DOEdesing of experiments”; 2.4.1. “Particle size, size distribution and zeta potential”; 2.4.2. “Morphology”. 2.4.3. “Amitriptyline encapsulation efficiency”; 2.5. “In vitro release assays”; (Pages: 6-10).

In vitro release assay: Additional information on the rationale behind the chosen parameters, such as the selection of PBS (pH 7.4) and the duration of the experiment, could be provided.

R: As previously mentioned, additional information addressing these important questions has been included in the “In vitro release assays”, in the Methods section (Page: 9, lines: 200-209).

Animal studies: The justification for using different doses of Ami-S and Ami-NPs in the FST could be clarified.

R: The section 2.6.2 “*Description of the Forced Swimming Test, doses of Ami-S and Ami-NPs used, and control solutions”* in the Method section has been modified to clarify the different doses used in Ami-NPs and Ami-S, and the rationale behind their selection (Page: 11, lines: 239-255).

Including details on estrous cycle determination methods strengthens the research.

R: A new section has been added in the Method section (2.6.1.	“Determination of estrous cycle phase”). This section describes the details about the cytological analyses performed to the female rats to determine the estrous phase (Page: 10, lines: 221-236).

6. PLOS authors have the option to publish the peer review history of their article ([what does this mean?](https://c05y1x9s.r.us-east-2.awstrack.me/L0/https:%2F%2Fjournals.plos.org%2Fplosone%2Fs%2Feditorial-and-peer-review-process%23loc-peer-review-history/1/010f01919044e900-41fe1632-c19e-4c7c-8f82-38dc44f61c55-000000/r9wb7wJ24DmErF7-W_mkItqk6yM=173)). If published, this will include your full peer review and any attached files.


If you choose “no”, your identity will remain anonymous but your review may still be made public.


**Do you want your identity to be public for this peer review?** For information about this choice, including consent withdrawal, please see our [Privacy Policy](https://c05y1x9s.r.us-east-2.awstrack.me/L0/https:%2F%2Fwww.plos.org%2Fprivacy-policy/1/010f01919044e900-41fe1632-c19e-4c7c-8f82-38dc44f61c55-000000/369TOuekjBRJ7jvx1rhp-bzD9d4=173).

Reviewer #1: **Yes:** Dr. Raja Chakraverty

Reviewer #2: **Yes:** Bharat Kumar Reddy Sanapalli

[NOTE: If reviewer comments were submitted as an attachment file, they will be attached to this email and accessible via the submission site. Please log into your account, locate the manuscript record, and check for the action link "View Attachments". If this link does not appear, there are no attachment files.]

While revising your submission, please upload your figure files to the Preflight Analysis and Conversion Engine (PACE) digital diagnostic tool, [https://pacev2.apexcovantage.com/](https://c05y1x9s.r.us-east-2.awstrack.me/L0/https:%2F%2Fpacev2.apexcovantage.com%2F/1/010f01919044e900-41fe1632-c19e-4c7c-8f82-38dc44f61c55-000000/4aHJdN6s3B7s96YbkqcIXOJpnfg=173). PACE helps ensure that figures meet PLOS requirements. To use PACE, you must first register as a user. Registration is free. Then, login and navigate to the UPLOAD tab, where you will find detailed instructions on how to use the tool. If you encounter any issues or have any questions when using PACE, please email PLOS at [figures@plos.org](mailto:figures@plos.org). Please note that Supporting Information files do not need this step.

**Reference**

Kedia, S. & Chattarji, S. (2014) Marble burying as a test of the delayed anxiogenic effects of acute immobilisation stress in mice. *J Neurosci Methods*, **233**, 150-154.
